# Analyses of Menopause and Its Related Symptoms on Sleep Quality Using a Novel Wearable Sheet-Type Frontal Electroencephalography Sensor, Haru-1

**DOI:** 10.1089/whr.2025.0007

**Published:** 2025-04-10

**Authors:** Kenjiro Sawada, Taro Yagi, Yizhi Liu, Shusuke Yoshimoto, Masaki Kobayashi, Kotaro Shimura, Misa Yamamoto, Gaku Yamamoto, Michiko Kodama, Hirohisa Kurachi, Tsuyoshi Sekitani, Tadashi Kimura

**Affiliations:** ^1^ Department of Obstetrics and Gynecology, Osaka University Graduate School of Medicine, Osaka, Japan.; ^2^ Department of Obstetrics and Gynecology, Osaka General Medical Center, Osaka, Japan.; ^3^ PGV Inc., Tokyo, Japan.; ^4^ Sakura Ladies’ Clinic, Osaka, Japan.; ^5^ Shimura Women’s Clinic, Osaka, Japan.; ^6^ Kansai Rosai Hospital, Hyogo, Japan.; ^7^ Osaka Women’s and Children’s Hospital, Osaka, Japan.; ^8^ The Institute of Scientific and Industrial Research, Osaka University, Osaka, Japan.; ^9^ Sakai City Medical Center, Osaka, Japan.

**Keywords:** climacteric syndrome, sleeping disorder, Haru-1, wearable sheet-type frontal EEG sensors

## Abstract

**Objectives:**

Menopause affects sleep quality and contributes to depressive symptoms, but its precise impact on sleep remains unclear. To address this gap, we conducted detailed measurements of brain activity during sleep in pre- and postmenopausal women using a novel wearable sheet-type frontal electroencephalography (EEG) patch, Haru-1 (PGV Inc., Tokyo, Japan).

**Methods:**

Hospitalized patients aged 30–50 years who had undergone bilateral salpingo-oophorectomy, as well as volunteer participants aged 40–60 years who underwent EEG monitoring at home, were enrolled in the study. EEG recordings were obtained during sleep. Participants were assessed for menopausal symptoms using the Simple Menopausal Index (SMI) and for depressive symptoms using the Quick Inventory of Depressive Symptomatology Japanese version (QIDS-J). Sleep parameters were calculated to investigate the relationships between menopause, menopausal symptoms, depressive symptoms, and sleep characteristics.

**Results:**

A total of 174 participants were recruited, and data from 126 participants were included in the final analysis. Among 126 analyzed participants (mean age: 46.3 ± 7.3 years; 36 premenopausal, 90 postmenopausal), postmenopausal women had lower sleep efficiency (78% vs. 88%; *p* = 0.0065) and longer sleep onset latency (20 minutes vs. 8 minutes; *p* = 0.0203). The presence of menopausal symptoms (SMI ≥51) correlated with shorter deep sleep (9 minutes vs. 26 minutes; *p* = 0.0367), and depressive symptoms (QIDS-J ≥6) were associated with prolonged wake after sleep onset (56 minutes vs. 36 minutes; *p* = 0.0242).

**Conclusions:**

Menopause was associated with reduced sleep efficiency and increased sleep onset latency. Detailed EEG analyses may contribute to a better understanding of the pathogenesis of menopausal symptoms and their impact on sleep.

## Introduction

In Japan, the menopausal period is defined as the 10-year interval encompassing the 5 years before and after the cessation of menstrual cycles, typically between the ages of 45 and 55.^1^ Symptoms that emerge during this period and are not attributable to organic changes are referred to as menopausal symptoms. Among these, menopausal disorder describes the spectrum of symptoms that significantly interfere with daily life. Menopausal symptoms are diverse and are generally categorized into three groups: vasomotor symptoms, such as hot flashes and sweating; psychological symptoms, including insomnia, anxiety, and depression; and physical symptoms, such as headaches, lower back pain, joint pain, and skin dryness.^2^

Menopause is also well known to affect sleep, with menopause-related insomnia being linked to changes in circadian rhythms and vasomotor symptoms.^2,3^ For example, the Study of Women’s Health Across the Nation cross-sectional study reported that 37.3% of women aged 40–55 years experienced difficulty sleeping.^4^ Furthermore, approximately 15%–50% of perimenopausal and postmenopausal women may suffer from a range of psychological and emotional symptoms, including anxiety, depression, insomnia, and forgetfulness, all of which can contribute to sleep disorders.^5^

Sleep parameters analyzed *via* sleep electroencephalography (EEG) provide a preferred method for investigating sleep disorders. However, research specifically addressing sleep changes during menopause is limited.^³^ For instance, Lampio et al. conducted a longitudinal analysis using polysomnography (PSG) on 60 perimenopausal women, reporting a decrease in total sleep time (TST) and an increase in wake after sleep onset (WASO).^6^ Simplifying sleep EEG measurements, expanding their application to larger cohorts, performing detailed analyses, and identifying sleep patterns characteristic of menopause may elucidate the mechanisms underlying sleep disorders in menopausal women.

Conventional PSG analyses involve complex procedures, often requiring hospitalization. To address this, the Sekitani Laboratory (https://www.sekitani-lab.com/) at the Institute of Scientific and Industrial Research, Osaka University, in collaboration with PGV Inc., has developed a novel wearable sheet-type frontal EEG measurement device, Haru-1. This device enables seamless brain wave measurement with accuracy comparable to that of traditional EEG^7,8^ and has demonstrated a sleep stage scoring accuracy of 78.6% and an F1 score of 73.4%, comparable with conventional PSG devices.^9^

In this study, we applied this novel wearable EEG system to measure brain waves in postmenopausal women. By utilizing this patch EEG device, we aimed to investigate the effects of menopause and menopausal symptoms on sleep patterns.

## Methods

### Participants and study design

This study was approved by the Osaka University Hospital Ethics Committee (approval number: 18021-6) and conducted in accordance with the ethical standards of the Declaration of Helsinki (revised 2013). Funding was provided by the Japan Agency for Medical Research and Development (AMED) Project for Whole Implementation to Support and Ensure the Female Life (WISE) (research number 20gk0210022h0002; 2019–2021).

The primary objective was to collect information on unique EEG patterns associated with menopause and examine whether menopausal and depressive symptoms affect sleep quality. To this end, participants were selected from patients experiencing sudden, inevitable loss of ovarian function due to gynecological surgeries. Inpatients were chosen to facilitate overnight monitoring, allowing researchers familiar with the EEG device to attend to participants as needed. Patients receiving treatments that could affect mental status, such as oral antianxiety drugs, sleeping pills, hormone replacement therapy, traditional Chinese medicine, steroids, anticancer drugs, or radiation therapy, were excluded. Furthermore, a medical history interview was conducted before the enrollment, and individuals with conditions that affect sleep, such as sleep apnea syndromes or restless legs syndrome, were excluded.

Participants included women aged 30–50 years who were admitted to the gynecological ward of Osaka University Hospital between April 2019 and March 2022. Eligible patients underwent bilateral salpingo-oophorectomy or more extensive gynecological surgery. EEG recordings were obtained using a patch-type measurement sheet (Haru-1) attached to the forehead of consenting participants before they fell asleep. Recordings were conducted immediately prior to hospital discharge (approximately one week after surgery) to minimize the intrinsic effects of the invasive procedure.

For the control group, consenting patients undergoing surgery for benign gynecological diseases without bilateral ovariectomy were similarly evaluated for EEG recordings prior to hospital discharge. Additionally, from April 2021 to July 2021, healthy women aged 40–60 years participated in EEG measurements and completed questionnaires at home. Written informed consent was obtained from all participants. In general, the first night in an unfamiliar environment is characterized by reduced sleep quality and alterations in sleep architecture.^10^ To minimize the impact of this so-called first-night effect (FNE), measurements were conducted on 2 consecutive days whenever possible, and data from the second day were used if measurements from both days were available.

Climacteric symptoms were assessed using the Simple Menopausal Index (SMI; Supplementary Table S1).^11^ The SMI is a 10-question scale commonly used in Japan that evaluates vasomotor, psychological, and somatic symptoms, rated on a 4-point scale (none, mild, moderate, severe). Scores range from 0 to 100, with the following classifications: ≤26 points (no symptoms), 26–50 points (mild), 51–65 points (moderate), and ≥66 points (severe). In this study, the cutoff score for moderate symptoms was set at 51.^12,13^

Depressive symptoms were assessed using the Japanese version of the Quick Inventory of Depressive Symptomatology (QIDS-J; Supplementary Table S2), which is derived from the validated QIDS–Self Report (QIDS-SR). This scale includes 16 questions across nine categories: 4 questions on sleep disorders, 4 on appetite and body weight changes, 2 on psychological symptoms, and 6 on other depressive symptoms. Scores were calculated by summing the highest scores from relevant domains. Participants with scores ≥6 were classified as having depression.^14,15^

### EEG device and parameters

**FIG. 1.**
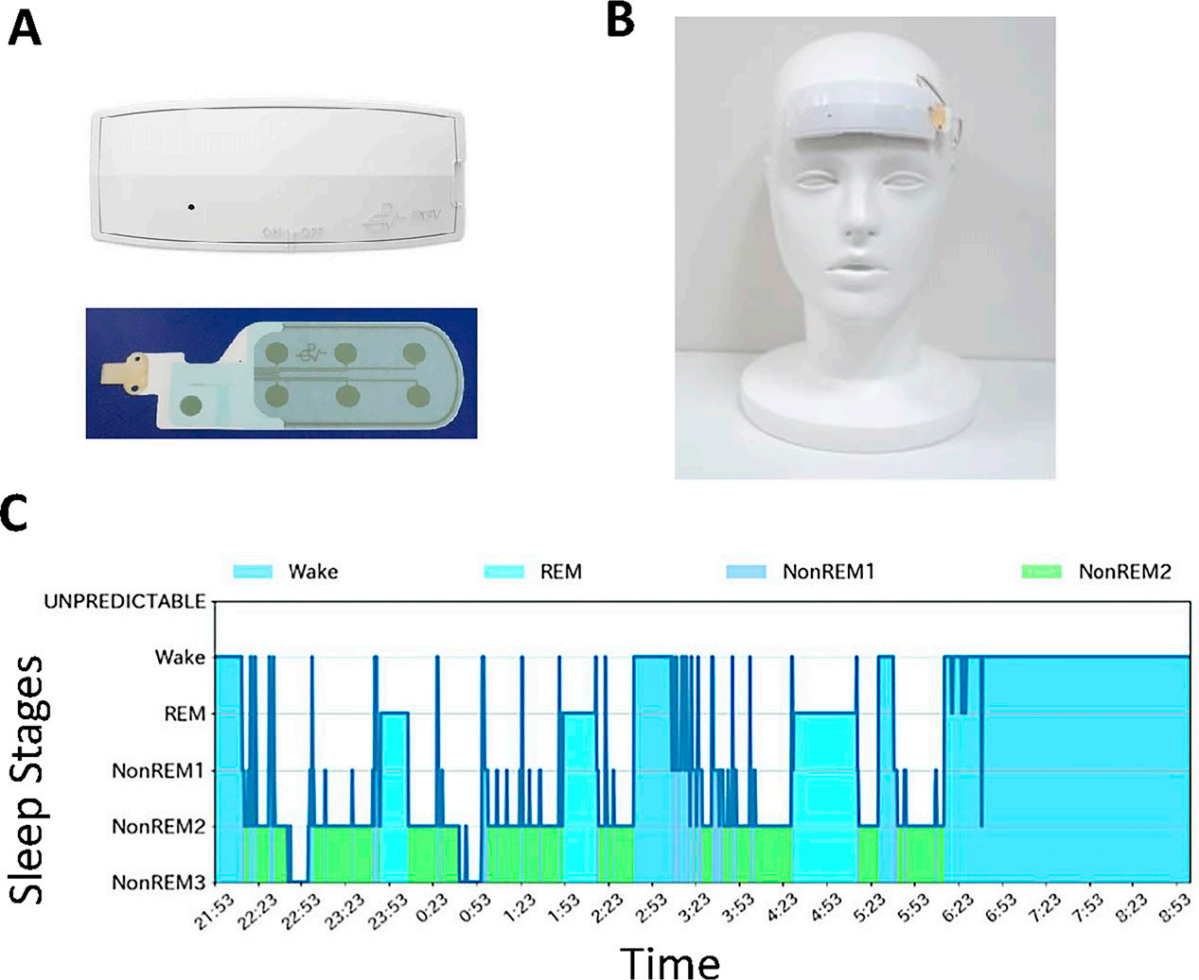
Sleep analyses using a novel wearable sheet-type frontal electroencephalography (EEG) sensor, Haru-1. **(A)** Upper, Haru-1. Lower, a patch sheet. **(B)** A mannequin wearing a patch sheet Haru-1. **(C)** An example of a sleep analysis. The vertical axis represents the depth of sleep (with lower values representing deeper sleep), and the horizontal axis represents time.

The wearable patch EEG (Haru-1; Fig. 1A), developed by the Sekitani Laboratory at the Institute of Scientific and Industrial Research, Osaka University, and commercialized by PGV Inc. (Tokyo, Japan), is a certified medical device (“telemetric EEG,” Class II; certification number: 302AFBZX00079000) in Japan. The device is lightweight (27 g), easy to install *via* a stretchable electrode sheet, and wirelessly controlled using data terminals such as tablets. Its low manufacturing cost ensures accessibility. The device utilizes noise processing technology to achieve highly accurate brain wave assessments. EEG data are recorded by attaching the patch to the forehead (Fig. 1B).^7,8^

Figure 1C illustrates an example of brain wave data obtained using the patch EEG. The following sleep parameters were analyzed:

- Time in bed (TIB): Total time spent in bed.
- TST: Duration from sleep onset to the final awakening, excluding wake periods.
- Sleep period time (SPT): Time from sleep onset to final awakening.
- Sleep efficiency (SE): TST divided by TIB, expressed as a percentage.
- Sleep onset latency (SOL): Time from bedtime to sleep onset.
- Deep sleep latency (SON3P): Interval from sleep onset to the first N3 (non-rapid eye movement [REM] deep sleep) period.
- WASO: Total wake time during the sleep period.
- Arousal times: Number of wake episodes after sleep onset.
- Arousal hour mean: Number of wake instances per hour.
- Stage shifts: Total number of sleep stage transitions.
- REM latency (REML): Interval from sleep onset to the first REM sleep period.
- REM sleep time: Total REM sleep duration.
- Durations of non-REM sleep stages (N1, N2, and N3).

### Statistical analyses

Statistical analyses were performed using JMP 17.0.0 (SAS Institute Inc., Cary, NC, USA). Sleep quality parameters were compared using the Wilcoxon rank-sum test. A *p* value of <0.05 was considered statistically significant.

## Results

**FIG. 2.**
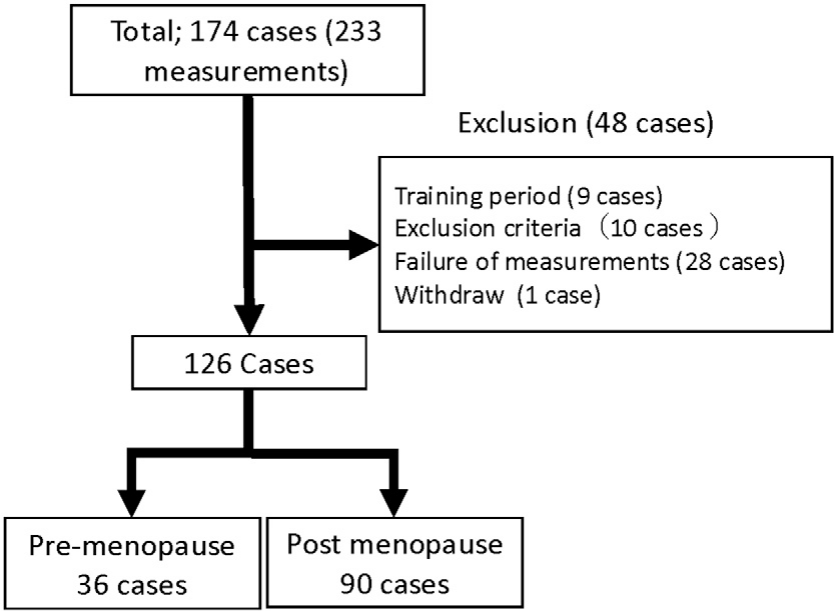
Flow chart of the measurements.

The flow chart of the patient inclusion process is shown in Figure 2. A total of 174 participants provided informed consent during the study period, and 233 EEG measurements were conducted. After excluding 48 cases due to measurement errors or other reasons, data from 126 cases were included in the final analysis. Of the 126 participants, 43 (34%) were able to undergo measurements on two consecutive days, and sleep data from the second day were used. The remaining 83 participants (67%; all inpatients) had scheduling constraints, such as discharge dates, which made it impossible to conduct measurements on both days. Therefore, data from the single available day were used. Of these, 36 cases (29%) were in the premenopausal group, and 90 cases (71%) were in the menopausal group, having undergone bilateral salpingo-oophorectomy or experienced natural menopause.

In this study, inpatients were selected from individuals who experienced a sudden and unavoidable loss of ovarian function due to gynecological surgeries. This selection criterion explains why inpatients with surgical menopause aged 30–50 years were included. In contrast, to analyze healthy volunteers (outpatients), we aimed to investigate the effects of natural menopause. Consequently, volunteer participants aged 40–60 years were included, as natural menopause typically occurs around the age of 50. As a result, the average age of the 13 outpatients was 49.5 ± 6.2 years, which was higher than that of the 113 inpatients (45.9 ± 7.3 years) (Supplementary Table S3). Furthermore, sleep parameters were compared between outpatients and inpatients (Table 1). According to hospital policy, inpatients were required to turn off the lights at 10:00 p.m. and get up at 6:00 a.m., resulting in a significantly longer TIB (477 minutes) compared to outpatients (345 minutes) (*p* < 0.0001). Consequently, TST and SPT were also significantly longer in inpatients. However, SE was significantly lower in inpatients (79%) than in outpatients (90%) (*p* = 0.029), and both SON3P and WASO were prolonged in inpatients. Meanwhile, deep sleep time (N3) was significantly longer in outpatients than in inpatients (50 minutes vs. 22 minutes; *p* = 0.0012).

**Table 1.** Sleep Parameters of the Participants Between Inpatients and Outpatients

	Inpatients (*n* = 113)	Outpatients (*n* = 13)	*p-*Values
Time in bed (TIB) (minutes)	477 (451–500)	345 (296–366)	<0.0001
Total sleep time (TST) (minutes)	375 (293–421)	283 (260–321)	0.0029
Sleep period time (SPT) (minutes)	439 (405–465)	310 (270–346)	<0.0001
Sleep efficacy (SE) (%)	79 (66–88)	90 (83–93)	0.029
Sleep onset latency (SOL) (minutes)	19 (6–33)	8 (6–17)	0.12
Deep sleeping latency (SON3P) (minutes)	40 (21–134)	19 (10–63)	0.014
Wake after sleep onset (WASO) (minutes)	51 (19–97)	16 (12–37)	0.0097
Arousal times (frequency)	19 (13–28)	12 (9–18)	0.011
Arousal hour mean (frequency)	3 (2–4)	2 (2–4)	0.33
Stage shifts (frequency)	60 (44–81)	35 (29–65)	0.0097
REM latency (REML)(minutes)	56 (3–91)	62 (1–99)	0.97
Rapid eye movement (REM) (minutes)	109 (71–136)	97 (74–114)	0.41
N1 (minutes)	25 (13–38)	12 (6–18)	0.0018
N2 (minutes)	192 (137–229)	123 (87–161)	0.001
N3 (minutes)	22 (4–42)	50 (30–66)	0.0012

Data are given as median (interquartile range [IQR]), analyzed with Wilcoxon rank-sum test.

Among the 90 menopausal participants, 86 were inpatients with iatrogenic menopause, and the remaining 4 were outpatients with natural menopause. Participant characteristics are summarized in Supplementary Table S4. The average age of the 4 participants with natural menopause was 56.0 ± 2.7 years, which was significantly higher than that of the 86 participants with iatrogenic menopause (47.0 ± 7.3 years; *p* = 0.0086). Sleep parameters were compared between the two groups (Table 2). Since participants with iatrogenic menopause were inpatients, while those with natural menopause were outpatients, both TIB and SPT were significantly longer in the iatrogenic menopause group (474 minutes vs. 317 minutes, and 432 minutes vs. 275 minutes, respectively). While significantly longer WASO was observed in patients with iatrogenic menopause compared with those with natural menopause (51 minutes vs. 15 minutes; *p* = 0.028), deep sleep time (N3) was significantly shorter in the iatrogenic menopause group than in the natural menopause group (21 minutes vs. 57 minutes; *p* = 0.016).

**Table 2.** Sleep Parameters of the Participants with Iatrogenic and Natural Menopause

	Iatrogenic (*n* = 86)	Natural (*n* = 4)	*p*-Values
Time in bed (TIB) (minutes)	474 (451–496)	317 (289–372)	0.0013
Total sleep time (TST) (minutes)	364 (291–408)	263 (258–292)	0.051
Sleep period time (SPT) (minutes)	432 (398–458)	275 (270–326)	0.005
Sleep efficacy (SE) (%)	78 (64–87)	89 (72–91)	0.19
Sleep onset latency (SOL) (minutes)	20 (8–34)	13 (4–30)	0.44
Deep sleeping latency (SON3P) (minutes)	41 (19–120)	30 (9–70)	0.37
Wake after sleep onset (WASO) (minutes)	51 (28–99)	15 (9–34)	0.028
Arousal times (frequency)	19 (14–27)	15 (10–20)	0.21
Arousal hour mean (frequency)	3 (2–4)	3 (2–4)	0.79
Stage shifts (frequency)	60 (44–76)	51 (34–67)	0.35
REM latency (REML)(minutes)	56 (7–97)	37 (0–116)	0.66
Rapid eye movement (REM) (minutes)	97 (59–133)	92 (47–112)	0.46
N1 (minutes)	25 (14–41)	12 (10–14)	0.0427
N2 (minutes)	181 (135–226)	106 (90–121)	0.013
N3 (minutes)	21 (4–40)	57 (50–103)	0.016

Data are given as median (IQR), analyzed with Wilcoxon rank-sum test.

Participant characteristics between the premenopausal group and menopausal group are summarized in Table 3. The mean age of premenopausal participants was 43.4 ± 6.1 years, while that of postmenopausal participants was 47.4 ± 7.4 years, a statistically significant difference. Patch EEG recordings were obtained from 113 participants (90%) during their hospital stay and from 13 participants (10%) at home. Among the 90 postmenopausal participants, 74 (82%) had undergone bilateral salpingo-oophorectomy or more extensive gynecological surgery. There were no significant differences between the two groups in questionnaire scores (SMI and QIDS-J).

**Table 3.** Characteristics of the Participants in the Premenopausal and Postmenopausal Groups

	Total [*n* = 126 (%)]	Premenopause (*n* = 36)	Postmenopause (*n* = 90)	*p* value
Age (years)	46.3 ± 7.3	43.4 ± 6.1	47.4 ± 7.4	0.0027
Body mass index (kg/m^2^)	22.4 ± 4.4	22.5 ± 4.1	22.3 ± 4.6	0.89
Inpatients	113 (90)	27	86	
Healthy volunteers	13 (10)	9	4	
Reason of menopause; *n* (%)		NA		
Bilateral Oophorectomy	74 (59)		74 (82)	
Pelvic irradiation	12 (9)		12 (13)	
Natural menopause	4 (3)		4 (5)	
SMI–median (IQR)	26 (18–40)	31 (16–42)	26 (20–38)	0.60
QIDS-J—median (IQR)	6 (4–9)	6 (3–9)	6 (4–10)	0.49

Age and BMI are presented as mean ± SD. Other data are presented as median (IQR), and *p*-values were analyzed using the Wilcoxon rank-sum test.

NA, not applicable; QIDS-J, Quick Inventory of Depressive Symptomatology Japanese version; SMI, Simple Menopausal Index.

To assess the influence of ovarian function on sleep, we compared sleep parameters between the premenopausal (36 participants) and postmenopausal groups (90 participants), as shown in Table 4. While there was no significant difference in TIB between the groups (469 minutes vs. 472 minutes), SE was significantly lower in the postmenopausal group (78% vs. 88%, *p* = 0.0065). SOL was also significantly longer in the postmenopausal group (20 minutes vs. 8 minutes, *p* = 0.0203). WASO tended to be longer in the postmenopausal group (50 minutes vs. 27 minutes, *p* = 0.0779), though the difference was not statistically significant. No significant differences were observed between the groups in other sleep stage parameters.

**Table 4.** Sleep Parameters Between Premenopausal Group and Postmenopausal Group

	Premenopause (*n* = 36)	Postmenopause (*n* = 90)	*p*-Values
Time in bed (TIB) (minutes)	469 (362–501)	472 (447–493)	0.44
Total sleep time (TST) (minutes)	366 (284–441)	361 (279–404)	0.35
Sleep period time (SPT) (minutes)	438 (350–473)	430 (380–456)	0.55
Sleep efficacy (SE) (%)	88 (75–94)	78 (65–88)	0.0065
Sleep onset latency (SOL) (minutes)	8 (4–21)	20 (8–34)	0.0203
Deep sleeping latency (SON3P) (minutes)	35 (19–154)	41 (17–119)	0.95
Wake after sleep onset (WASO) (minutes)	27 (12–79)	50 (26–93)	0.0779
Arousal times (frequency)	16 (8–26)	19 (14–27)	0.11
Arousal hour mean (frequency)	2 (1–3)	3 (2–4)	0.0307
Stage shifts (frequency)	59 (34–81)	60 (43–76)	0.44
REM latency (REML) (minutes)	58 (1–77)	56 (5–97)	0.73
Rapid eye movement (REM) (minutes)	112 (85–152)	97 (59–130)	0.0806
N1 (minutes)	23 (8–31)	24 (13–39)	0.14
N2 (minutes)	186 (114–232)	175 (127–224)	0.90
N3 (minutes)	30 (9–46)	22 (5–43)	0.29

Data are given as median (IQR), analyzed with Wilcoxon rank-sum test.

To evaluate the effect of menopausal symptoms on sleep, we compared participants with SMI scores ≥51 (18 participants) with those with SMI scores <51 (108 participants). Participant characteristics are presented in Supplementary Table S5. While there were no significant differences in age or body mass index (BMI), higher SMI scores were significantly associated with higher QIDS-J scores (*p* = 0.0194). Sleep analyses (Table 5) revealed that SPT tended to be shorter in patients with SMI scores ≥51 compared with those with scores <51 (411 minutes vs. 435 minutes, *p* = 0.092). Notably, N3 (deep sleep) duration was significantly shorter in participants with SMI scores ≥51 (9 minutes) compared with those with scores <51 (26 minutes, *p* = 0.0367).

**Table 5.** Sleep Parameters of the Participants with SMI ≥51 and SMI <51

	SMI ≥51 (*n* = 18)	SMI <51 (*n* = 108)	*p*-Values
Time in bed (TIB) (minutes)	470 (407–478)	471 (443–498)	0.43
Total sleep time (TST) (minutes)	329 (236–401)	364 (290–419)	0.15
Sleep period time (SPT) (minutes)	411 (306–444)	435 (378–464)	0.0922
Sleep efficacy (SE) (%)	76 (54–89)	82 (68–90)	0.36
Sleep onset latency (SOL) (minutes)	17 (3–80)	17 (6–32)	0.43
Deep sleeping latency (SON3P) (minutes)	65 (22–387)	38 (19–111)	0.32
Wake after sleep onset (WASO) (minutes)	44 (20–105)	49 (17–84)	0.82
Arousal times (frequency)	16 (10–23)	19 (12–27)	0.33
Arousal hour mean (frequency)	3 (2–4)	3 (2–4)	0.85
Stage shifts (frequency)	51 (37–67)	61 (42–80)	0.15
REM latency (REML)(minutes)	68 (1–139)	55 (3–81)	0.40
Rapid eye movement (REM) (minutes)	102 (49–129)	107 (74–138)	0.47
N1 (minutes)	27 (19–36)	23 (12–36)	0.35
N2 (minutes)	168 (127–229)	186 (123–226)	0.58
N3 (minutes)	9 (0–29)	26 (7–46)	0.0367

Data are given as median (IQR), analyzed with Wilcoxon rank-sum test.

Finally, the effect of depressive symptoms on sleep was analyzed based on QIDS-J scores. Participants with QIDS-J scores ≥6 (73 participants) were compared with those with scores <6 (53 participants), as shown in Supplementary Table S6. The mean age of participants with scores ≥6 was 45.2 ± 7.4 years, which was significantly younger than those with scores <6 (47.8 ± 6.8 years, *p* = 0.0388). Sleep parameter analysis (Table 6) showed that TIB was significantly longer in participants with QIDS-J scores ≥6 compared with those with scores <6 (479 minutes vs. 462 minutes, *p* = 0.0174). However, there were no significant differences in TST or SPT. WASO was significantly longer in participants with QIDS-J scores ≥6 (56 minutes vs. 36 minutes, *p* = 0.0242). Additionally, deep sleep latency was longer in participants with scores ≥6 (49 minutes vs. 28 minutes), although the difference was not statistically significant (*p* = 0.0631).

**Table 6.** Sleep Parameters of the Participants with QIDS-J ≥6 and QIDS-J <6

	QIDS-J ≥6 (*n* = 73)	QIDS-*J* <6 (*n* = 53)	*p*-Values
Time in bed (TIB) (minutes)	479 (445–504)	462 (387–480)	0.0174
Total sleep time (TST) (minutes)	363 (290–421)	357 (274–410)	0.66
Sleep period time (SPT) (minutes)	436 (395–466)	427 (337–461)	0.24
Sleep efficacy (SE) (%)	79 (63–88)	83 (68–91)	0.21
Sleep onset latency (SOL) (minutes)	18 (7–33)	16 (5–32)	0.56
Deep sleeping latency (SON3P) (minutes)	49 (22–129)	28 (16–94)	0.0631
Wake after sleep onset (WASO) (minutes)	56 (25–108)	36 (15–69)	0.0242
Arousal times (frequency)	19 (14–29)	17 (10–22)	0.0505
Arousal hour mean (frequency)	3 (2–4)	3 (2–4)	0.39
Stage shifts (frequency)	58 (45–75)	59 (36–83)	0.81
REM latency (REML)(minutes)	62 (15–115)	51 (2–74)	0.10
Rapid eye movement (REM) (minutes)	109 (69–135)	106 (78–134)	0.90
N1 (minutes)	23 (14–33)	25 (10–39)	1.00
N2 (minutes)	175 (135–226)	192 (107–227)	0.58
N3 (minutes)	22 (5–41)	31 (5–49)	0.49

Data are given as median (IQR), analyzed with Wilcoxon rank-sum test.

## Discussion

In this study, we utilized a novel patch EEG device, Haru-1, to measure sleep EEG in premenopausal and postmenopausal women. Our findings revealed that postmenopausal women exhibited lower SE and longer SOL compared with premenopausal women. Furthermore, participants with severe menopausal symptoms experienced a significantly shorter duration of deep sleep compared with those without severe menopausal symptoms. Additionally, participants with depressive symptoms demonstrated significantly prolonged WASO compared with those without depressive symptoms.

In the comparison of sleep parameters between outpatients and inpatients (Table 1), outpatients exhibited better SE, shorter SON3P, shorter WASO, and longer deep sleep time (N3). While inpatients were 30–50 years old with or without bilateral oophrectomy, volunteer participants aged 40–60 years were included. In addition to the sudden loss of ovarian function in inpatients, age differences and environmental differences may partly contribute to the significant differences in sleep parameters between outpatients and inpatients. Furthermore, in the comparison of sleep parameters between participants with iatrogenic menopause and those with natural menopause (Table 2), participants with iatrogenic menopause exhibited not only significantly longer WASO compared with those with natural menopause but also significantly shorter deep sleep time (N3). These findings indicate that participants with iatrogenic menopause experience poorer sleep quality compared to those with natural menopause. In addition to age and environmental differences, a possible reason for this discrepancy is that the iatrogenic menopause group includes patients with premature ovarian insufficiency (POI). Several studies have shown that women with POI are more likely to suffer from poor sleep quality, insomnia, and depression than healthy women.^16,17^ For instance, Huang et al. reported that among women with POI, the most prevalent symptoms were mood swings (73.4%), insomnia (58.7%), sexual problems (58.7%), and fatigue (57.3%).^17^ Accordingly, compared with women with natural menopause, women with POI exhibited a significantly higher risk of insomnia, with an odds ratio of 1.41 (95% confidence interval [CI]: 1.02–1.96).

The relationship between menopausal symptoms and sleep remains complex and inconclusive. While Freedman et al. reported that hot flashes do not contribute to sleep disturbances in postmenopausal women,^14,15^ other studies have suggested that menopausal symptoms decrease TST, increase WASO, and alter sleep brain waves, such as by increasing beta wave activity.^6,18^ Furthermore, a tri-directional relationship between menopausal symptoms, mood, and sleep disturbances has been reported in the menopausal transition.^3^ In a longitudinal analysis of 309 women transitioning menopause, depressive symptoms were unrelated to menopausal status or annual change in estradiol (E2) but were associated with hot flashes and sleep disturbance.^19^ Burleson et al. reported that daily vasomotor symptoms predicted same-day sleep problems and next-day positive mood, while sleep problems predicted worse mood on the next day.^20^ Due to the practical challenges of conducting large-scale analyses using conventional PSG, research on menopause-related changes in sleep brain waves has been limited.^3^ A notable strength of this study is its relatively large sample size (126 participants), which provides valuable insights into sleep disorders associated with menopause and related symptoms.

We also investigated the impact of menopausal symptoms on sleep using the SMI, a widely used scale in Japan. Deep sleep duration was significantly shorter in participants with SMI scores ≥51 compared to those with scores <51, suggesting that menopausal symptoms may impair the ability to achieve deep sleep.

In addition, differences in sleep parameters were analyzed based on the presence or absence of depressive symptoms. Participants with QIDS-J scores ≥6 exhibited significantly longer WASO and prolonged latency to deep sleep (SON3P), although the latter difference was not statistically significant. Previous studies on EEG in depressive patients have reported prolonged sleep latency, increased WASO, early morning awakenings, shortened REM latency, increased REM sleep, and decreased deep sleep.^7,21,22^ Similar trends were observed in this study, reinforcing the association between depressive symptoms and changes in sleep architecture.

This study has several limitations. First, the study was conducted at a single institution, with all participants being Japanese. Since menopausal symptoms and sleep disorders vary depending on racial and cultural backgrounds, careful interpretation of the results is required. Second, while we intended to analyze the effects of menopause on both sleep parameters and participants’ moods, we only collected data on menopausal symptoms (SMI) and depressive symptoms (QIDS-J). We did not obtain specific data on insomnia disorder and chronic insomnia, although we excluded participants who were taking sleeping pills. To accurately assess the efficacy of Haru-1 for diagnosing insomnia disorder or chronic insomnia, subjective sleep measures such as the Pittsburgh Sleep Quality Index would be indispensable. Third, in sleep analysis, it is recommended to conduct measurements over two or more consecutive days to minimize the FNE. However, due to scheduling constraints, such as participants’ discharge dates, sleep data were only collected on the first day of measurement for 83 of the 113 inpatients. Consequently, the recorded sleep quality may have been poorer than the actual baseline. Fourth, most participants were hospitalized for gynecological conditions, and EEG measurements were conducted at least one week after surgery, immediately before discharge. As a result, the sample may have included individuals who were not yet experiencing typical menopausal symptoms. Additionally, hospitalization and surgery could have negatively impacted mental status, potentially leading to elevated QIDS-J scores. To address these limitations, future studies should include home-based measurements in a general perimenopausal population to validate the findings and broaden the applicability of this method. We are currently developing a research plan to explore this avenue.

## Conclusion

This study, utilizing the novel sheet-type EEG patch Haru-1, demonstrated that postmenopausal women experience lower SE and prolonged SOL. Furthermore, menopausal symptoms were associated with reduced deep sleep duration, while depressive symptoms were linked to significantly longer WASO. Sleep brain wave analyses with Haru-1 enabled detailed assessment of sleep quality changes in perimenopausal women. Introducing this simple and objective method into clinical practice has the potential to improve understanding of menopausal symptoms and enhance the quality of clinical care.

## Data Availability

Data used in this study are available upon request from the corresponding author.

## Abbreviations Used

EEG: Electroencephalography
POI: Premature ovarian insufficiency
PSG: Polysomnography
QIDS-J: Quick Inventory of Depressive Symptomatology Japanese version
REML: REM Latency
SE: Sleep Efficiency
SMI: Simple Menopausal Index
SOL: Sleep Onset Latency
SON3P: Deep Sleep Latency
SPT: Sleep Period Time
SWAN: Study of Women's Health Across the Nation
TIB: Time in Bed
TST: Total Sleep Time
WASO: Wake after sleep onset

## References

[1] Japan Society of Obstetrics and Gynecology. Glossary of obstetrics and gynecology ver. 4. Japan Society of Obstetrics and Gynecology; 2018, pp. 74–75.

[2] Monteleone P, Mascagni G, Giannini A, et al. Symptoms of menopause—Global prevalence, physiology and implications. Nat Rev Endocrinol 2018;14(4):199–215; doi: 10.1038/nrendo.2017.180 29393299

[3] Baker FC, Lampio L, Saaresranta T, et al. Sleep and sleep disorders in the menopausal transition. Sleep Med Clin 2018;13(3):443–456; doi: 10.1016/j.jsmc.2018.04.011 30098758 PMC6092036

[4] Gold EB, Sternfeld B, Kelsey JL, et al. Relation of demographic and lifestyle factors to symptoms in a multi-racial/ethnic population of women 40-55 years of age. Am J Epidemiol 2000;152(5):463–473; doi: 10.1093/aje/152.5.463 10981461

[5] Huang S, Wang Z, Zheng D, et al. Anxiety disorder in menopausal women and the intervention efficacy of mindfulness-based stress reduction. Am J Transl Res 2023;15(3):2016–2024. 37056841 PMC10086901

[6] Lampio L, Polo-Kantola P, Himanen S-L, et al. Sleep during menopausal transition: A 6-year follow-up. Sleep 2017;40(7); doi: 10.1093/sleep/zsx090 28525646

[7] Araki T, Uemura T, Yoshimoto S, et al. Wireless monitoring using a stretchable and transparent sensor sheet containing metal nanowires. Adv Mater 2020;32(15):e1902684; doi: 10.1002/adma.201902684 31782576

[8] Araki T, Yoshimoto S, Uemura T, et al. Skin-like transparent sensor sheet for remote healthcare using electroencephalography and photoplethysmography. Adv Materials Technologies 2022;7(11):2200362; doi: 10.1002/admt.202200362

[9] Matsumori S, Teramoto K, Iyori H, et al. HARU sleep: A deep learning-based sleep scoring system with wearable sheet-type frontal EEG sensors. IEEE Access 2022;10(4):13624–13632; doi: 10.1109/ACCESS.2022.3146337

[10] Wick AZ, Combertaldi SL, Rasch B. The first-night effect of sleep occurs over nonconsecutive nights in unfamiliar and familiar environments. Sleep 2024;47(10):zsae179; doi: 10.1093/sleep/zsae179 39126649 PMC11467056

[11] Koyama T. Background and interpretation of simplified menopausal index. J Jpn Menopause Soc 1998;6:93. (in Japanese).

[12] Suka M, Taniuchi A, Kudo Y, et al. Self-assessed health and menopausal symptoms among 50-year-old Japanese women: Cross-sectional surveys in Northern Kawasaki in 1998 and Menopause 2010;17(1):166–173; doi: 10.1097/gme.0b013e3181b6683f 19724241

[13] Kai Y, Nagamatsu T, Kitabatake Y, et al. Effects of stretching on menopausal and depressive symptoms in middle-aged women: A randomized controlled trial. Menopause 2016;23(8):827–832; doi: 10.1097/GME.0000000000000651 27300113 PMC4961267

[14] Kessler RC, Berglund P, Demler O, et al. Lifetime prevalence and age-of-onset distributions of DSM-IV disorders in the National Comorbidity Survey Replication. Arch Gen Psychiatry 2005;62(6):593–602; doi: 10.1001/archpsyc.62.6.593 15939837

[15] Freedman RR, Roehrs TA. Lack of sleep disturbance from menopausal hot flashes. Fertil Steril 2004;82(1):138–144; doi: 10.1016/j.fertnstert.2003.12.029 15237002

[16] Ates S, Aydın S, Ozcan P, et al. Sleep, depression, anxiety and fatigue in women with premature ovarian insufficiency. J Psychosom Obstet Gynaecol 2022;43(4):482–487; doi: 10.1080/0167482X.2022.2069008 35531877

[17] Huang Y, Qi T, Ma L, et al. Menopausal symptoms in women with premature ovarian insufficiency: Prevalence, severity, and associated factors. Menopause 2021;28(5):529–537; doi: 10.1097/GME.0000000000001733 33470756

[18] Kravitz HM, Avery E, Sowers M, et al. Relationships between menopausal and mood symptoms and EEG sleep measures in a multi-ethnic sample of middle-aged women: The SWAN sleep study. Sleep 2011;34(9):1221–1232; doi: 10.5665/SLEEP.1244 21886360 PMC3157664

[19] Avis NE, Crawford S, Stellato R, et al. Longitudinal study of hormone levels and depression among women transitioning through menopause. Climacteric 2001;4(3):243–249. 11588948

[20] Burleson MH, Todd M, Trevathan WR. Daily vasomotor symptoms, sleep problems, and mood: Using daily data to evaluate the domino hypothesis in middle-aged women. Menopause 2010;17(1):87–95; doi: 10.1097/gme.0b013e3181b20b2d 19675506

[21] Steiger A, Pawlowski M. Depression and sleep. Int J Mol Sci 2019;20(3):607; doi: 10.3390/ijms20030607 30708948 PMC6386825

[22] Ding L, Zhang L, Cui Y, et al. The association of sleep duration and quality with depressive symptoms in older Chinese women. PLoS One 2022;17(3):e0262331; doi: 10.1371/journal.pone.0262331 35290372 PMC8923433

