# Supplementary material for: Analyses of Menopause and Its Related Symptoms on Sleep Quality Using a Novel Wearable Sheet-Type Frontal Electroencephalography Sensor, Haru-1
Source: Womens Health Rep (New Rochelle). 2025 Apr 10;6(1):393–402. doi: 10.1089/whr.2025.0007 (PMC12040546; doi:10.1089/whr.2025.0007)
Supplement: Supplementary Table S1 [file whr.2025.0007_supplementary_table_s1.docx]

| Symptoms | Severe | Moderate | Mild | Absent |
| --- | --- | --- | --- | --- |
| Hot flashes | 10 | 6 | 3 | 0 |
| Sweats | 10 | 6 | 3 | 0 |
| Cold constitution | 14 | 9 | 5 | 0 |
| Shortness of breath or palpitation | 12 | 8 | 4 | 0 |
| Insomnia | 14 | 9 | 5 | 0 |
| Easy excitability or irritability | 12 | 8 | 4 | 0 |
| Worry about self depression | 7 | 5 | 3 | 0 |
| Headache, vertigo or nausea | 7 | 5 | 3 | 0 |
| Easy fatigability | 7 | 4 | 2 | 0 |
| Shoulder stiffness, lumbago or joint pain | 7 | 5 | 3 | 0 |

**Supplementary Table 1.** Simple Menopausal Index (SMI)
