# Supplementary material for: Analyses of Menopause and Its Related Symptoms on Sleep Quality Using a Novel Wearable Sheet-Type Frontal Electroencephalography Sensor, Haru-1
Source: Womens Health Rep (New Rochelle). 2025 Apr 10;6(1):393–402. doi: 10.1089/whr.2025.0007 (PMC12040546; doi:10.1089/whr.2025.0007)
Supplement: Supplementary Table S3 [file whr.2025.0007_supplementary_table_s3.docx]

|  | Inpatients (N=113) | Outpatients (N=13) | P-values |
| --- | --- | --- | --- |
| Age (years) | 49.5 ± 6.2 | 45.9 ± 7.3 | 0.10 |
| Body mass index (kg/m^2^) | 22.4 ± 4.6 | 21.6 ± 4.4 | 0.64 |
| Reason of menopause; n (%) |  |  | <0.0001 |
| Bilateral Oophorectomy | 74 (65) | 0 (0) |  |
| Pelvic irradiation | 12 (11) | 0 (0) |  |
| Natural menopause | 0 (0) | 4 (31) |  |
| Premenopause | 27 (24) | 9 (69) |  |
| SMI - median (IQR) | 26 (16-36) | 40 (25-52) | 0.025 |
| QIDS-J - median (IQR) | 6 (4-10) | 5 (2-8) | 0.052 |
| SMI; Simple Menopausal Index, QIDS-J; Quick Inventory of Depressive Symptomatology Japanese version | | | |

**Supplementary Table 3.** Characteristics of the participants between inpatients and outpatients. Age and BMI are presented as mean ± SD. Other data are presented as median (IQR), and p-values were analyzed using the Wilcoxon rank-sum test.
