# Supplementary material for: Analyses of Menopause and Its Related Symptoms on Sleep Quality Using a Novel Wearable Sheet-Type Frontal Electroencephalography Sensor, Haru-1
Source: Womens Health Rep (New Rochelle). 2025 Apr 10;6(1):393–402. doi: 10.1089/whr.2025.0007 (PMC12040546; doi:10.1089/whr.2025.0007)
Supplement: Supplementary Table S4 [file whr.2025.0007_supplementary_table_s4.docx]

|  | Iatrogenic (N=86) | Natural  (N=4) | P-values |
| --- | --- | --- | --- |
| Age (years) | 47.0 ± 7.3 | 56.0 ± 2.7 | 0.0086 |
| Body mass index (kg/m^2^) | 22.4 ± 4.6 | 19.5 ± 1.6 | 0.31 |
| Reason of menopause; n (%) |  |  |  |
| Bilateral Oophorectomy | 74 | 0 |  |
| Pelvic irradiation | 12 | 0 |  |
| Natural menopause | 0 | 4 |  |
| SMI - median (IQR) | 26 (20-38) | 32 (11-41) | 0.91 |
| QIDS-J - median (IQR) | 7 (4-10) | 3 (1-6) | 0.059 |

**Supplementary Table 4.** Characteristics of the participants with iatrogenic and natural menopause. Age and BMI are presented as mean ± SD. Other data are presented as median (IQR), and p-values were analyzed using the Wilcoxon rank-sum test.
