# Supplementary material for: Analyses of Menopause and Its Related Symptoms on Sleep Quality Using a Novel Wearable Sheet-Type Frontal Electroencephalography Sensor, Haru-1
Source: Womens Health Rep (New Rochelle). 2025 Apr 10;6(1):393–402. doi: 10.1089/whr.2025.0007 (PMC12040546; doi:10.1089/whr.2025.0007)
Supplement: Supplementary Table S5 [file whr.2025.0007_supplementary_table_s5.docx]

|  | SMI ≥ 51 (N=18) | SMI < 51 (N=108) | P-value |
| --- | --- | --- | --- |
| Age (years) | 47.9 ± 8.3 | 46.0 ± 7.1 | 0.36 |
| Body mass index (kg/m^2^) | 21.5 ± 3.7 | 22.5 ± 4.5 | 0.39 |
| Reason of menopause; n (%) |  |  | 0.74 |
| Bilateral Oophorectomy | 12 (67) | 62 (57) |  |
| Pelvic irradiation | 2 (11) | 10 (9) |  |
| Natural menopause | 0 (0) | 4 (4) |  |
| Premenopause | 4 (22) | 32 (30) |  |
| QIDS-J - median (IQR) | 8 (6-13) | 6 (4-9) | 0.0194 |

**Supplementary Table 5.** Characteristics of the participants with SMI ≥ 51 and KI < 51. Age and BMI are presented as mean ± SD. Other data are presented as median (IQR), and p-values were analyzed using the Wilcoxon rank-sum test.
