# Supplementary material for: Analyses of Menopause and Its Related Symptoms on Sleep Quality Using a Novel Wearable Sheet-Type Frontal Electroencephalography Sensor, Haru-1
Source: Womens Health Rep (New Rochelle). 2025 Apr 10;6(1):393–402. doi: 10.1089/whr.2025.0007 (PMC12040546; doi:10.1089/whr.2025.0007)
Supplement: Supplementary Table S6 [file whr.2025.0007_supplementary_table_s6.docx]

|  | QIDS-J ≥ 6 (N=73) | QIDS-J < 6 (N=53) | P-values |
| --- | --- | --- | --- |
| Age (years) | 45.2 ± 7.4 | 47.8 ± 6.8 | 0.0388 |
| Body mass index (kg/m^2^) | 22.5 ± 4.2 | 22.2 ± 4.8 | 0.69 |
| Reason of menopause; n (%) |  |  | 0.54 |
| Bilateral Oophorectomy | 43 (59) | 31 (58) |  |
| Pelvic irradiation | 8 (11) | 4 (8) |  |
| Natural menopause | 1 (1) | 3 (6) |  |
| Premenopause | 21 (29) | 15 (28) |  |
| SMI - median (IQR) | 30 (23-44) | 22 (13-35) | 0.0032 |

**Supplementary Table 6.** Characteristics of the participants with QIDS-J ≥ 6 and QIDS-J < 6. Age and BMI are presented as mean ± SD. Other data are presented as median (IQR), and p-values were analyzed using the Wilcoxon rank-sum test.
